# Reproductive-dependent effects of B vitamin deficiency on lifespan and physiology

**DOI:** 10.3389/fnut.2023.1277715

**Published:** 2023-10-24

**Authors:** Guixiang Yu, Shaowei Liu, Kun Yang, Qi Wu

**Affiliations:** ^1^Key Laboratory for Aging and Regenerative Medicine, Department of Pharmacology, School of Pharmacy, Southwest Medical University, Luzhou, Sichuan, China; ^2^Central Nervous System Drug Key Laboratory of Sichuan Province, Luzhou, Sichuan, China

**Keywords:** B vitamin, *Drosophila*, lifespan, reproduction, fat metabolism

## Abstract

B vitamins constitute essential micronutrients in animal organisms, executing crucial roles in numerous biological processes. B vitamin deficiency can result in severe health consequences, including the impairment of reproductive functions and increased susceptibility to age-related diseases. However, the understanding of how reproduction alters the requirements of each individual B vitamins for healthy aging and lifespan remains limited. Here, utilizing *Drosophila* as a model organism, we revealed the substantial impacts of deficiencies in specific B vitamins on lifespan and diverse physiological functions, with the effects being significantly shaped by reproductive status. Notably, the dietary absence of VB_1_, VB_3_, VB_5_, VB_6_, or VB_7_ significantly decreased the lifespan of wild-type females, yet demonstrated relatively little effect on *ovo^*D*1^* infertile mutant females’ lifespan. B vitamin deficiencies also resulted in distinct impacts on the reproduction, starvation tolerance and fat metabolism of wild-type females, though no apparent effects were observed in the infertile mutant females. Moreover, a deficiency in VB_1_ reshaped the impacts of macronutrient intervention on the physiology and lifespan of fertile females in a reproductive-dependent manner. Overall, our study unravels that the reproductive status of females serves as a critical modulator of the lifespan and physiological alterations elicited by B-vitamin deficiencies.

## 1. Introduction

Vitamins, a pivotal class of micronutrients, are mainly obtained through diet and are crucial for the preservation of normal physiological functions among animals. To date, thirteen distinct vitamins have been identified, inclusive of vitamins A, C, D, E, K, and an assembly of eight water-soluble B vitamins including Thiamine (B_1_), Riboflavin (B_2_), Niacin (B_3_), Pantothenic Acid (B_5_), Pyridoxine (B_6_), Biotin (B_7_), Folate (B_9_), and Cobalamin (B_12_) (1).

Vitamins play an instrumental role in facilitating diverse biological functions including growth, metabolism, development, and the promotion of healthy aging in animals. For instance, vitamin A is essential for vision and immune function, as well as skin and bone health. Vitamins C and E, which are potent antioxidants, hold paramount significance in tissue growth and repair. Vitamin D is necessary for calcium absorption and promoting bone growth whereas vitamin K plays a crucial role in blood clotting and maintaining bone health (1). Studies with model organisms suggest that adequate vitamin intake might attenuate the aging process and increase lifespan. Dietary supplementation with vitamin A has been shown to extend lifespan in *Drosophila* and rodent models such as mice (2, 3). Vitamin C supplementation increased lifespan in worms, mice and rats (4, 5). Furthermore, vitamin C also enhanced starvation resistance and increased the activity of antioxidant-related enzymes in *Drosophila* (6, 7). The nutritional addition of vitamin E has similarly been associated with an extension of lifespan in a range of organisms, ranging from worms and *Drosophila* to several rodent species (8). In addition, studies in centenarians also suggest a potential link between enhanced longevity and elevated plasma levels of vitamins A and E (9).

Similar to other classes of vitamins, B vitamins also serve indispensable roles in maintaining health and fitness in animal species (10, 11). These vitamins function variously as coenzymes, precursors, or substrates, playing a critical role in diverse biological processes such as energy metabolism, DNA damage repair and epigenetic regulation. A deficiency of B vitamins can induce a range of pathologies, from neurological deficits and immunological disorders to oncological conditions (11, 12). Moreover, B vitamin deficiency also detrimentally impacts the reproductive performance in species spanning insects to mammals, leading to hormone dysregulation, impaired germ cell development, and elevated risks of miscarriage and fetal anomalies (13–15). In humans, due to age-associated degradation in digestion, absorption, and alterations in diet and metabolism, the deficiency of B vitamins is more common within the elderly (16, 17). Despite recognized associations between B vitamin deficiencies and age-related diseases including neurodegenerative disorders, cardiovascular disease, and osteoporosis (18), the implications of various B vitamin deficiencies on aging, as well as how reproduction changes the requirements of each individual B vitamins for lifespan, remain elusive.

Multiple animal models have been used to study the effects of B vitamins on nutritional metabolism and diseases. Among them, the employment of the fruit fly (*Drosophila melanogaster*) as an experimental model offers a significant opportunity to delineate the consequences of B vitamin deficiency on aging, due to its short life-cycle, analogous fundamental nutritional requirements to humans, and adjustable dietary nutrient composition facilitated by well-established, chemically defined medium. Notably, the abundant easy-to-observe aging phenotypes of *Drosophila* enable meticulous observation. Earlier studies have demonstrated seven of the eight B vitamins, excluding B_12_, are the only group of vitamins requisite for the normal development of fruit fly larvae (19–22), but how these vitamins affect lifespan is not fully understood. In previous studies, the effects of thiamine and biotin deficiency on lifespan have been reported in *Drosophila*. In *Canton S* strain, biotin deficiency in adult diet decreased lifespan (23), but thiamine deficiency had no significant effect on lifespan in both conventionally reared or axenic conditions (24). Dietary supplementation of riboflavin significantly improved the survival and oxidative stress resistance in *Oregon K* line flies (25). Our previous studies found that in conventionally reared condition, removing all B vitamins from the medium significantly shortened the lifespan of wild-type *Dahomey* females, but had little effect on the lifespan of males, which at least partially due to nutrient competition for female reproduction (26). Here, we further investigated the effects of individual B vitamins on the lifespan and physiology of female fruit flies, and revealed the effects of reproduction on the B vitamin requirements for female lifespan.

## 2. Materials and methods

### 2.1. Fly stock and husbandry

The wild-type stock *Dahomey* were gift from Yang Lab (17). BL1,309 (ovo[D1] v[24]/C(1)DX, y[1] w[1] f[1]) were obtained from the Bloomington Stock Center and backcrossed into wild-type *Dahomey* for eight generations. *ovo^*D*1^* males were crossed with *Dahomey* females to obtain sterile *Dah, ovo^*D*1^* females. All stocks were maintained at 25°C on a 12-h: 12-h light:dark cycle at 60% humidity using 1SY food [7 g/L agar; 50 g/L sucrose; 100 g/L yeast (Yeast brand: ANGEL YA100)]. For all experiments, flies were reared at standard larval density in 1SY food and eclosed adults were collected over a 12-h period (27). Flies were mated for 48 h on 1SY food in all experiments before sorting into single sexes. Forty-eight hour-mated females were collected and transferred to FLYaa holidic medium [(28); Supplementary Table 1] for lifespan and all physiology experiment and maintained at 25°C on a 12-h:12-h light:dark cycle at 60% humidity. The vitamin concentration [thiamine (Sigma-Aldrich, T4625) = 1.4 mg/L; riboflavin (Sigma-Aldrich, R4500) = 0.7 mg/L; nicotinic acid (Sigma-Aldrich, N4126) = 8.4 mg/L; Ca pantothenate (Sigma-Aldrich, P21210) = 10.8 mg/L; pyridoxine (Sigma-Aldrich, P9755) = 1.7 mg/L; biotin (Sigma-Aldrich, B4501) = 0.1 mg/L; and folic acid (Sigma-Aldrich, F7876) = 0.5 mg/L] used in the FLYaa medium in this experiment is consistent with previous described (22, 28), since it optimized the development time of fruit flies and the egg-laying performance of adult flies. 100N50S FLYaa (amino acids concentration = 100 mM; sucrose concentration = 50 mM) diet was used as control diet.

### 2.2. Lifespan analysis

**FIGURE 1:**
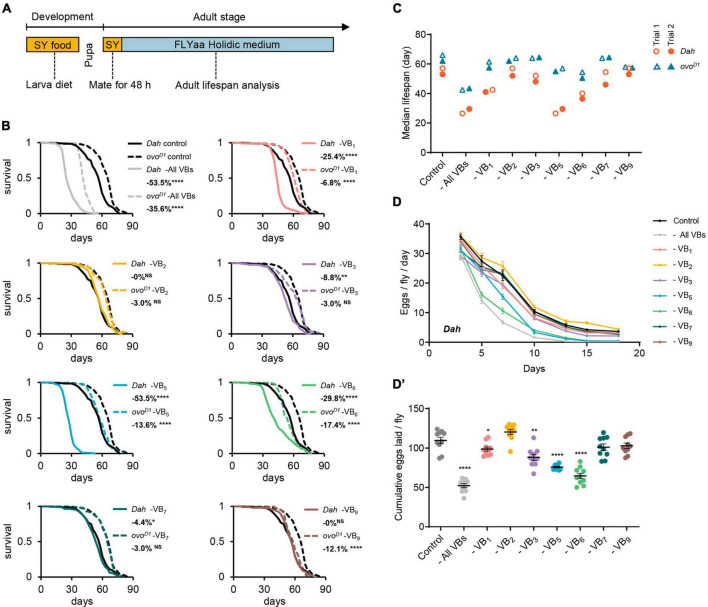
Effect of B vitamins deficiency on lifespan and fecundity in females. **(A)** Experimental timeline for rearing and exposure of adult flies to experimental diets. Effect of omitting all B vitamins or individual B vitamin on lifespan curves **(B)** and median lifespan **(C)** of wild-type *Dahomey* females and *ovo^*D*1^* mutant females. Effect of omitting all B vitamins or individual B vitamin on daily egg production **(D)** and cumulative eggs laid **(D’)** of wild-type *Dahomey* females. For lifespan experiments in panel **(B)**, 200 wild-type *Dahomey* flies and 100 *ovo^*D*1^* mutant female flies were used for each treatment. The median lifespan data of panel **(C)** were from 2 independent batches of lifespan experiments, in which the median lifespan data of Trial 1 correspond to the lifespan curves of panel **(B)**, and the median lifespan data of Trial 2 correspond to the lifespan curves of Supplementary Figure 1. *n* = 10 biological replicates for egg laying in all trials, error bars represent egg-laying mean ± SEM. (Within each phenotype, *p < 0.05, **p < 0.01, ****p < 0.0001 versus complete 100N50S FLYaa diet control. Lifespan differences were assessed using the log-rank test, and egg-laying differences were assessed by one-way ANOVA followed by Tukey’s multiple comparison. See statistical analysis of lifespan data in Supplementary Tables 2, 3.)

Flies were randomly allocated to the experimental food treatments and housed in plastic vials containing food at a density of 10 flies per vial, with 10 vials per condition (100 flies were used for each treatment) for all lifespan experiments except Figure 1B. In Figure 1B, lifespan experiment of *Dahomey* female flies was performed with 20 vials (10 flies per vial) per treatment. Flies were transferred to a fresh food source every 2–3 days, during which any deaths and censors were recorded. Lifespan differences were assessed using the log-rank test.

### 2.3. Fecundity

The number of eggs laid in 24-h periods were counted every 2–3 days (flies were transferred to fresh experimental food at ZT0-ZT1, the number of eggs laid were counted at ZT0-ZT1 the next day), and data are reported as cumulative eggs laid per female in the results. For each condition and each time point, 10 vials were counted. Each vial contained 10 flies. Egg-laying differences were assessed by one-way ANOVA followed by Tukey’s multiple comparison.

### 2.4. EX-Q feeding assay

Food intake was measured using the excreta quantification (EX-Q) feeding assay developed by us (29). Briefly, flies fed with experimental FLYaa diets for 14 days (10 flies/vial, with 10 vials/treatment) were transferred from FLYaa medium to EX-Q vials with dye labeled FLYaa food (diameter = 5.5 mm) contained 0.2% Erioglaucine disodium salt (Sigma-Aldrich, 861146) and kept for 24 h. The excreta were collected and dissolved in 2 ml 0.1% PBST, the absorbance of the liquid sample was then measured at 630 nm (Molecular Devices, FlexStation 3) and used for food intake calculation.

### 2.5. Body weight measurement

Flies fed with experimental FLYaa diets for 14 days were anesthetized with CO_2_ and collected into 1.5 ml Eppendorf tubes for body weight measurement. For each treatment, 12 tubes were measured, each tube contained 10 flies. Data were assessed using the unpaired *t*-test and reported as average body weight per female in the figures.

### 2.6. Starvation assay

Flies fed with experimental diets for 14 days (10 flies/vial, with 10 vials/treatment) were transferred to vials containing only 0.7% agar to provide moisture. Deaths were recorded every day. Replaced fresh agar food every 3 days. Survival was assessed using the log-rank test.

### 2.7. Ovary size measurement

Flies fed with experimental diets for 14 days were anesthetized with ice and dissected in PBS to obtain ovaries. Ovaries were fixed in 4% formaldehyde/PBS for 1 h and mounted in Antifade mounting media (Vector H-1200). Quantification of the ovary size was performed using ImageJ.

### 2.8. Triacylglyceride assay

Flies fed with experimental diets for 14 days (10 flies/vial, with 12 vials/treatment) were weighed, then frozen in liquid nitrogen for triacylglyceride content quantification by using Triglyceride assay kit (Nanjing Jiancheng Bioengineering Institute, A110-1-1).

### 2.9. Gut Oil Red O staining

Gut Oil Red O (ORO) staining was performed as previously described (30). Briefly, flies fed with experimental diets for 14 days were anesthetized with ice and dissected in PBS to obtain guts. Guts were fixed in 4% formaldehyde/PBS for 30 min, then washed twice with PBS and incubated in a 0.5% ORO solution (solvent = 60% isopropanol + 40% ddH_2_O, ORO solution was filtered with a 0.45 μm syringe filter) for 30 min. After that, guts were washed twice with ddH_2_O and mounted in Antifade mounting media (Vector H-1200). Quantification of the ORO was performed using ImageJ.

## 3. Results

### 3.1. Reproductive-dependent effects of B vitamin deficiency on lifespan

To investigate the impact of B vitamin deficiency on the aging of female flies, we utilized FLYaa chemically defined medium (28) as the base medium for examining the effects of removing individual or all B vitamins on lifespan. To ensure the normal development of experimental flies and maintain consistent physiological conditions across all experimental groups, we fed the flies a sucrose/yeast medium (consisting of 5% sucrose and 10% yeast) from the egg to adult. Once the adult flies emerged, they were transferred to the same sucrose/yeast medium for a 48-h mating period before the females were isolated for lifespan and fecundity assessments on the 100N50S FLYaa medium (Figure 1A and Supplementary Table 1).

Consistent with our previous studies (26), the removal of all B vitamins from the adult diet substantially decreased the lifespan of female flies. Compared to the *ovo^*D*1^* infertile mutant females, the wild-type females exhibited an even greater extent of lifespan reduction. When individual B vitamin was deprived in the medium, both wild-type females and *ovo^*D*1^* mutant fruit flies showed varying degrees of lifespan reduction. The removal of VB_1_, VB_3_, VB_5_, or VB_6_ from the diet resulted in a decreased lifespan and fecundity for wild-type females; while VB_7_ deficiency caused only a minor decrease in lifespan with insignificant fertility impact (Figures 1B–D’ and Supplementary Figure 1). In contrast, the absence of VB_2_ or VB_9_ did not lead to noticeable lifespan changes in wild-type females (Figures 1B, C). VB_1_, VB_5_, or VB_6_ deficiency also shortened the lifespan of *ovo^*D*1^* mutant females, albeit with a smaller magnitude than that observed in the wild-type females. Interestingly, although the deprivation of VB_9_ did not affect the lifespan of wild-type females, it did shorten the lifespan of *ovo^*D*1^* mutant females (Figures 1B, C).

The impact of single vitamin deficiencies on lifespan demonstrates varying degrees of reproduction-dependency. Most notably, the removal of either VB_5_ or VB_6_ substantially diminished the reproductive capacity of wild-type females (Figures 1D, D’). However, in comparison to the impact of VB_6_ deficiency on the lifespan of wild-type females, the life-shortening effect under VB_5_ deficiency conditions is markedly more pronounced (Figures 1B, C). It suggests that the substantial decrease of lifespan under VB_5_ deficiency is largely attributable to nutritional competition mediated by reproduction. This is further substantiated by the observation that under conditions of VB_5_ or VB_6_ deprivation, the alterations in the lifespan of the infertile *ovo^*D*1^* mutant females is essentially identical (Figures 1B, C).

Furthermore, in wild-type females, VB_5_ deprivation resulted in comparable lifespan reductions to those observed when all vitamins were collectively removed (Figures 1B, C), suggesting that VB_5_ is the key factor limiting female lifespan under the condition of depriving all B vitamins. Contrasting with the “barrel effect” exhibited by wild-type female lifespan linked to VB_5_, the lifespan of *ovo^*D*1^* mutant displayed a cumulative decrease with multiple vitamin deficiencies.

### 3.2. B vitamin deficiency impacts the physiology of *Drosophila* in a reproductively dependent manner

**FIGURE 2:**
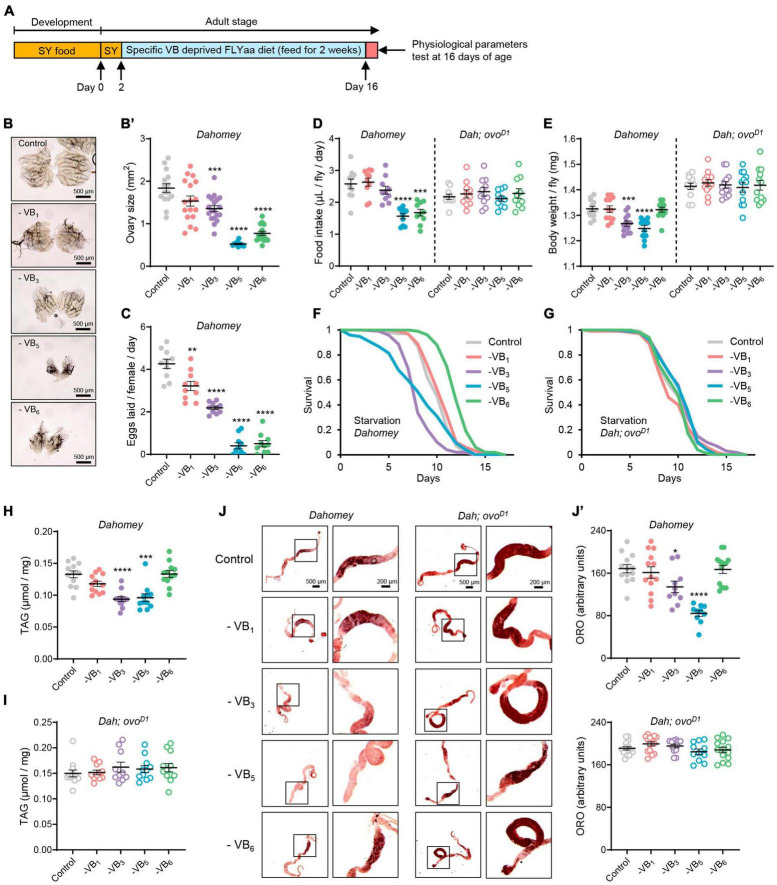
Reproduction dependent impacts of B vitamin deprivation on physiology of *Drosophila*. **(A)** Experimental timeline for dietary vitamin deprivation and physiological parameters tests. Effects of depriving B vitamins from 100N50S FLYaa diet for a duration of 14 days on ovary morphology **(B)**, ovary size **(B’)**, and egg laying **(C)** in wild-type *Dahomey* females. *n* = 11–19 ovaries for ovary size measurement; *n* = 10 biological replicates for egg laying count, with 10 flies per replicate; effects of depriving B vitamins from 100N50S FLYaa diet for a duration of 14 days on food intake **(D)**, body weight **(E)**, starvation resistance **(F,G)**, total triglyceride content **(H,I)**, and Oil Red O staining of midguts **(J,J’)** in wild-type *Dahomey* females and *ovo^*D*1^* mutant females. *n* = 10 biological replicates for food intake experiment, with 10 flies per replicate. A total of 100 flies were used per treatment for starvation experiment; *n* = 12 biological replicates for body weight and triglyceride measurement, with 10 flies per replicate; *n* = 10–14 guts per condition for quantification of Oil Red O staining. (Within each phenotype, *p < 0.05, **p < 0.01, ***p < 0.001, ****p < 0.0001 versus complete 100N50S FLYaa diet control. All data were assessed using the unpaired t test except for the starvation experiments of panels **(F,G)**. In panel **(F,G)**, *p*-values were determined by log-rank test.)

Given the significant increase in mortality of wild-type females after 2–3 weeks of vitamin B complex deprivation (Figures 1B, C), to reflect the alterations in female health during the initial stages of B vitamin deficiency, we examined the impact of a 14-day deprivation of vitamin B_1_, B_3_, B_5_, and B_6_ on female physiology (Figure 2A). Deprivation of VB_3_, VB_5_, or VB_6_ from the diet for a duration of 2 weeks results in significant reduction in the ovarian size of wild-type females, consistent with their effect on egg production (Figures 2B–C). The *ovo^*D*1^* infertile mutant females exhibited noticeable ovarian atrophy, and the removal of either VB_1_, VB_3_, VB_5_, or VB_6_ did not inflict substantial alterations on their ovarian morphology (Supplementary Figures 2A, A’). Given that reproductive state profoundly affects the nutritional requirements and appetites of animals (31, 32), we further examined the effect of B vitamin deprivation on food intake in both wild-type females and *ovo^*D*1^* mutant females. Indeed, the deficiency of either VB_5_ or VB_6_, which are crucial for reproduction, significantly decreased food intake in wild-type females. This observation formed a stark contrast to the *ovo^*D*1^* mutant females’ response, where no discernible shifts in food intake were detected (Figure 2D). Deprivation of VB_3_ or VB_5_ led to a significant weight drop in wild-type females. However, the removal of VB_6_ from diet failed to affect the body weight of fertile females, despite a concurrent decrease in reproductive output and food consumption (Figure 2E).

In addition to the effects on body weight, the deprivation of VB_3_ or VB_5_ also significantly reduced triglyceride levels in fertile females and increased their vulnerability to starvation (Figures 2F, H). Surprisingly, removing VB_6_ not only failed to decrease the starvation tolerance of fertile females but also substantially increased their survival time under starvation conditions (Figure 2F). This observation can be attributed to the fact that in the absence of VB_6_, although fecundity is significantly reduced, flies still maintained a relatively stable body weight and triglyceride content (Figures 2F, H), thus alleviating the competition between reproduction and somatic maintenance. In contrast to fertile females, the body weight, triglyceride content, and starvation resistance of *ovo^*D*1^* sterile females were unaffected by B vitamin deprivation (Figures 2E, G, I). Considering the crucial role the gut plays in lipid absorption, synthesis, storage, and transport in *Drosophila* (30, 33, 34), we assessed the gut-specific fat metabolism regulation by B vitamins by monitoring midgut fat accumulation under various B vitamin deficiency scenarios. Consistent with the effect on total triglyceride content, among the four vitamins (VB_1_, VB_3_, VB_5_, and VB_6_), only the deprivation of VB_3_ or VB_5_ significantly reduced fat content in the anterior midgut of wild-type females. Furthermore, B vitamin deficiency had no significant impact on intestinal fat content in *ovo^*D*1^* mutant females (Figures 2J, J’). These results suggest that reproduction exacerbates the physiological detriments induced by B vitamin deficiencies in females.

### 3.3. VB_1_ modifies the impacts of the relative abundance of macronutrients on longevity and physiology

The relative abundance of macronutrients in food plays a crucial role in determining the reproductive capacity and lifespan of animals (26, 35–37). Moreover, given that B vitamins are typically involved in sugar and amino acid metabolism as cofactors (38), an increase in the consumption of these macronutrients may expedite the depletion of B vitamins in cases where there is a deficiency. Our results illustrate that among the seven B vitamins tested, deficiencies in VB_1_, VB_5_, and VB_6_ exert most substantial impacts on the lifespan of females (Figure 1B). However, due to the ubiquity of VB_5_ in most conventional diets, its deficiency is rarely observed, compared to the more prevalent deficiencies in VB_1_ and VB_6_ (16). As the lifespan decrease caused by VB_1_ deficiency demonstrates a stronger reproductive dependence than that of VB_6_ (Figure 1B), we further investigated the potential impact of relative proportions of sugar and amino acids on the consequences of VB_1_ deficiency on lifespan and physiology.

**FIGURE 3:**
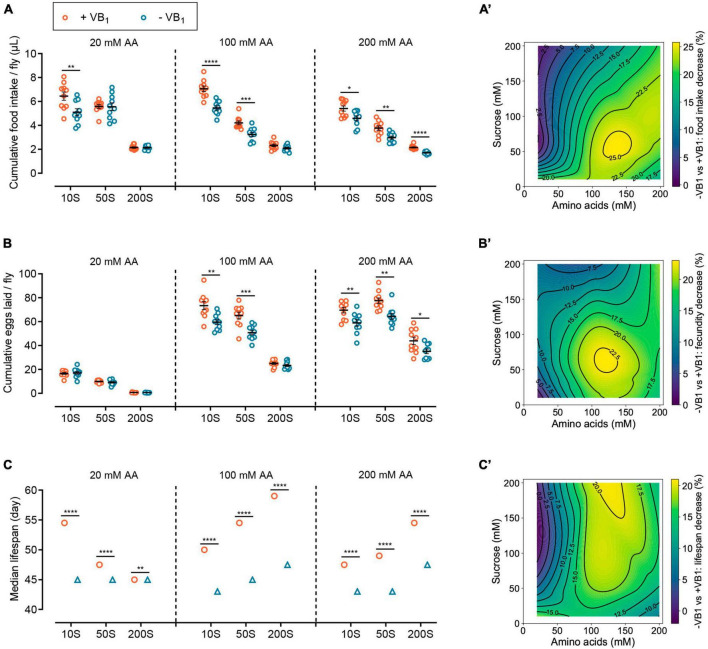
VB_1_ reshapes the impacts of the relative abundance of dietary sucrose and amino acids on lifespan and physiology. Effects of VB_1_ deficiency on cumulative food intake **(A)**, cumulative eggs laid **(B)**, and median lifespan **(C)** of wild-type *Dahomey* females across diets with varying sucrose or amino acid concentrations. The horizontal coordinates of 10S, 50S, and 200S represent sucrose concentration of 10, 50, and 200 mM in FLYaa diets, respectively. *n* = 10 biological replicates for food intake measurement and egg laying count, with 10 flies per replicate. The median lifespan data of panel **(C)** correspond to the lifespan curves of Supplementary Figure 3D, lifespan differences were assessed using log rank test. A total of 100 flies were used per treatment. See statistical analysis of lifespan data in Supplementary Table 4. Within each phenotype, *p < 0.05, **p < 0.01, ***p < 0.001, ****p < 0.0001 versus VB_1_-containing FLYaa diet control. Error bars represent mean ± SEM. Food intake and egg-laying data were assessed using the unpaired t test. VB_1_ deficiency caused decrease in cumulative food intake **(A’)**, cumulative eggs laid **(B’)**, and median lifespan **(C’)** were plotted against amino acids and sucrose content of nine diets by using contour and contourf in matplotlib.pyplot package of Python 3.10. Heat maps from dark green to yellow represent the percentage decline from lowest to highest.

Previous study has shown under the feeding conditions of 100N50S FLYaa medium, female flies exhibit both a relatively long lifespan and good reproductive performance (28). Therefore, using the 100N50S FLYaa medium as a control, we examined the effects of VB_1_ deficiency on female food intake, fecundity, and lifespan across nine different diets with varying sucrose or amino acid contents (Supplementary Figure 3A and Supplementary Table 1). The impact of VB_1_ deficiency on the food intake of wild-type females reveals a distinct age-dependent relation. In the early stages of VB_1_ deficiency, the food intake of females showed no significant changes compared to the control group fed with VB_1_-containing diet (Figure 2D). However, after 30 days of age, the decline in feeding caused by VB_1_ deficiency became evident under specific dietary conditions (Supplementary Figure 3B). Therefore, to effectively characterize the long-term effect of VB_1_ deficiency on food consumption, we compared the cumulative food intake of wild-type females at 10, 20, 30, and 40 days of age (Figure 3A). The results indicate that the impact of VB_1_ deficiency on food intake is closely related to the abundance of sugar and amino acids in the medium. In diets with low amino acid levels (20 mM) and moderate amino acid levels (100 mM), an increase in sucrose concentration mitigated the adverse effects of VB_1_ deficiency on food intake. However, under high amino acids (200 mM) dietary conditions, altering the sucrose content in the medium did not effectively alleviate the decline in feeding caused by VB_1_ deficiency (Figures 3A, A’). The variations in reproduction followed a similar pattern to that of food intake under different nutritional conditions. In diets rich in amino acids, VB_1_ deficiency resulted in a significant decline in egg production of wild-type females (Figures 3B, B’ and Supplementary Figure 3C). It suggests that under VB_1_-restricted conditions, the vigorous reproductive capacity caused by a high amino acid diet might exacerbate the depletion of stored VB_1_ in females, thereby amplifying the negative effects of VB_1_ deficiency. Conversely, increasing dietary sucrose reduces egg production by decreasing food consumption, thereby alleviating the competition for limited VB_1_, thus effectively mitigating the negative effects of VB_1_ deficiency.

VB_1_ deprivation also altered the response of female lifespan to variations in sugar and amino acid content in the diet. Overall, VB_1_ deficiency diminished the plasticity of female lifespan with regards to changes in sucrose and amino acid levels in the diet, particularly under low amino acid dietary conditions where VB_1_ deficiency entirely abolished the response of female lifespan to changes in sugar concentration. Moreover, under conditions of moderate amino acid levels and high sucrose diet, VB_1_ deficiency resulted in the most substantial decrease in lifespan (Figures 3C, C’ and Supplementary Figure 3D). Taken together, these results indicate that VB_1_ reshaped the impact of variations in the abundance of sugar and amino acids in the diet on female lifespan and physiology. Therefore, ensuring adequate VB_1_ intake plays a crucial role in enhancing the impact of dietary macronutrient interventions on longevity.

## 4. Discussion

**FIGURE 4:**
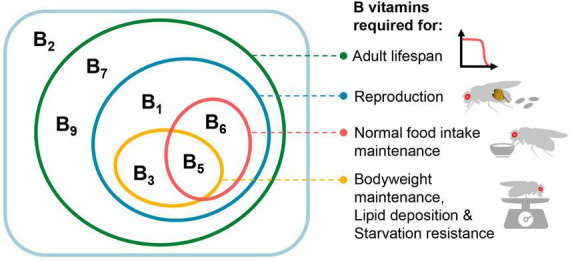
Effects of various B vitamins on the physiology of female *Drosophila melanogaster*. In the rectangular box are the seven B vitamins tested in this study (VB_12_ was not tested), with the effects of vitamins on different physiological activities represented by ellipses of different colors. For the effects on food intake, bodyweight, lipid deposition, and starvation resistance, only VB_1_, VB_3_, VB_5_, and VB_6_ were tested.

Together, our data shed light on the impact of dietary deficiencies of individual B vitamins on the lifespan and physiology of adult female *Drosophila*, as well as the reproductive dependence of these effects. Impacts of various B vitamin deficiencies manifest differently in female physiology (Figure 4). Among the four vitamins that significantly affect fecundity (VB_1_, VB_3_, VB_5_, and VB_6_), only VB_5_ and VB_6_ significantly reduced food intake. In contrast, VB_3_ does not alter female food intake but notably decreases body mass and lipid deposition levels. This may imply that a deficiency in VB_3_ potentially attenuates the efficiency of macronutrient absorption and conversion, which is consistent with its cofactor role in multiple intermediate steps involved in carbohydrate, amino acid and lipid metabolism (39). In the present study, we limited our investigations to the physiology changes of females during the initial stages of B vitamin deficiency. It is reasonable to conjecture that the adverse implications on physiology could exacerbate, should the period of deficiency prolong.

Overall, the detrimental impact of B vitamin deficiencies on the lifespan of infertile mutant females is less severe than on wild-type females, with the exception of VB_9_. This result underscores the significance of the competition for B vitamin between reproductive processes and somatic maintenance as a crucial determinant of female lifespan under conditions of vitamin B deficiency. The differential effects on lifespan and physiological functions caused by the deficiency of various B vitamins likely partially stem from differences in their half-lives within the body. Previous studies have shown that in humans, VB_1_, VB_2_, VB_3_, VB_5_, VB_6_, and VB_7_ are typically depleted within 1–4 weeks under restricted B vitamin conditions, while VB_9_ depletion typically occurs over weeks to months, and the depletion of VB_12_ can take several months or even more than a year (40, 41). This may explain why the deprivation of VB_9_ did not shorten the lifespan of wild-type females but slightly decreased the lifespan of longer-lived *ovo^*D*1^* mutant females, as wild-type females likely have reached their maximum lifespan before VB_9_ depletion occurs. It is worth noting that we exclusively examined the impact of varying vitamin deficiencies on fly lifespan under conventionally reared conditions, but did not investigate the case of axenic feeding. Considering that *Drosophila*-associated bacteria can synthesize and provide diverse B vitamins to flies (14, 24, 42, 43), deficiency of B vitamins under sterile feeding conditions may exert more severe effects on lifespan. In our study, VB_2_ deficiency had no significant effect on the lifespan of either wild-type females or *ovo^D1^* infertile females, suggesting that riboflavin acquired by flies during development and the first 2 days of adult stage, as well as from symbiotic bacteria, is sufficient to maintain full length of life.

Furthermore, the genetic characteristics and backgrounds of *Drosophila* likely play a notable role in determining lifespan under conditions of vitamin deficiency. While previous research suggests that the *Canton S* strain experiences no significant impact on lifespan in the context of vitamin B_1_ deficiency (24), our findings reveal a striking reduction in lifespan for *Dahomey* females deprived of vitamin B_1_. Correspondingly, our earlier study also noted dissimilarities in the decrease in lifespan between the *Canton S* and *Dahomey* strains when deprived of all B vitamins in the medium, with the *Canton S* strain exhibiting a smaller decline (26). This discrepancy could potentially stem from variations in the vitamin requirements for reproduction between the two strains.

Our study also presents novel evidence highlighting that micronutrients can modulate the response of animal lifespan and physiology to fluctuations in macronutrient abundance. Previous studies suggest that altering the dietary concentrations of macronutrients – notably sugars and amino acids – can effectively regulate animal lifespan (36, 37, 44). However, the impact of variations in these energy nutrients abundance in food on animal lifespan and physiology may not exclusively originate from these constituents’ direct metabolic effects. Instead, they may also stem from shifts in reproductive potential prompted by alterations in macronutrient intake, resulting in reallocation of non-energy nutrients between reproduction and lifespan maintenance. Recent study suggested that lifespan shortening associated with high-protein diets can, at least partially, be due to the overconsumption of limited cholesterol resources driven by intensive reproductive activities (45). Our study has also identified a similar interaction pattern between the micronutrient VB_1_ and sugar as well as amino acids. Under VB_1_ deficiency conditions, the increase in egg production caused by a high amino acid diet may accelerate the depletion of VB_1_, thereby exacerbating metabolic disorders and the frailty in females, leading to a cascade of adverse outcomes such as diminished appetite. Therefore, interventions in dietary levels of sugar and amino acids could potentially modulate the severity of deleterious physiological effects induced by VB_1_ deficiency through the regulation of reproduction. Furthermore, with adequate VB_1_ in the diet, variations in sugar and amino acid concentrations substantially affect female lifespan; however, under VB_1_-deficient conditions, the amplitude of lifespan variations caused by changing sugar and amino acid availability is considerably reduced. Given the pivotal function of VB_1_ as a coenzyme in sugar and amino acid metabolism (38), metabolic disorders and pathological changes caused by VB_1_ deficiency might also limit lifespan plasticity facilitated by dietary interventions controlling macronutrient intake. Considering the common decrease in appetite and food intake during the aging process, appropriate exogenous supplementation of B vitamins in the elderly will contribute to maximizing the effectiveness of anti-aging strategies based on macronutrient intake interventions.

## Data availability statement

The original contributions presented in this study are included in this article/Supplementary material, further inquiries can be directed to the corresponding author.

## Ethics statement

The manuscript presents research on animals that do not require ethical approval for their study.

## Author contributions

GY: Conceptualization, Data curation, Investigation, Methodology, Writing – review and editing, Writing – original draft. SL: Data curation, Investigation, Writing – review and editing. KY: Data curation, Investigation, Writing – review and editing. QW: Data curation, Investigation, Writing – review and editing, Conceptualization, Funding acquisition, Methodology, Project administration, Supervision.
