# Supplementary material for: Reproductive-dependent effects of B vitamin deficiency on lifespan and physiology
Source: Front Nutr. 2023 Oct 24;10:1277715. doi: 10.3389/fnut.2023.1277715 (PMC10627837; doi:10.3389/fnut.2023.1277715)
Supplement: Supplementary file 1 [file Data_Sheet_1.PDF]

Supplementary Material

1 Supplementary Figures and Tables

1.1 Supplementary Figures

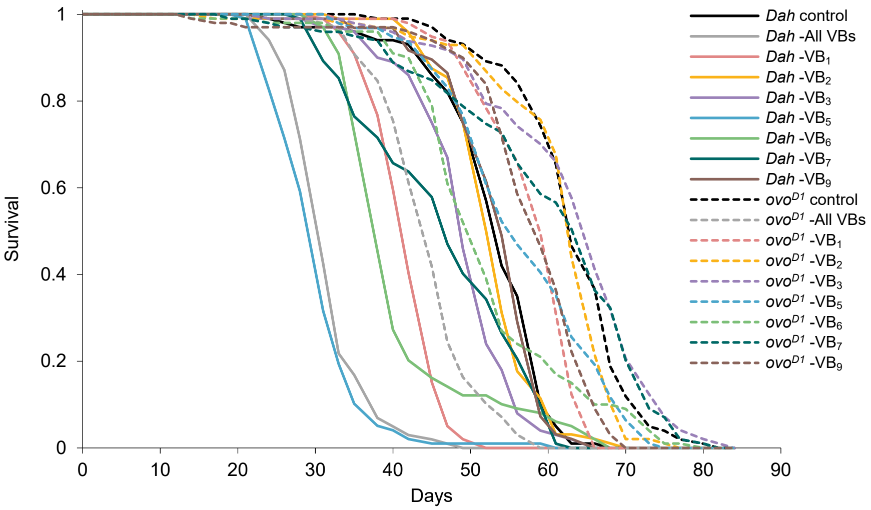

Supplementary Figure 1. Effect of B vitamins deficiency on lifespan in wild-type *Dahomey* females and *ovo*^{D1} mutant females. (These lifespan curves were related to the median lifespan data of trial 2 in Figure 1C. 100 flies were used per treatment. See statistical analysis of lifespan data in Supplementary Table 3.)

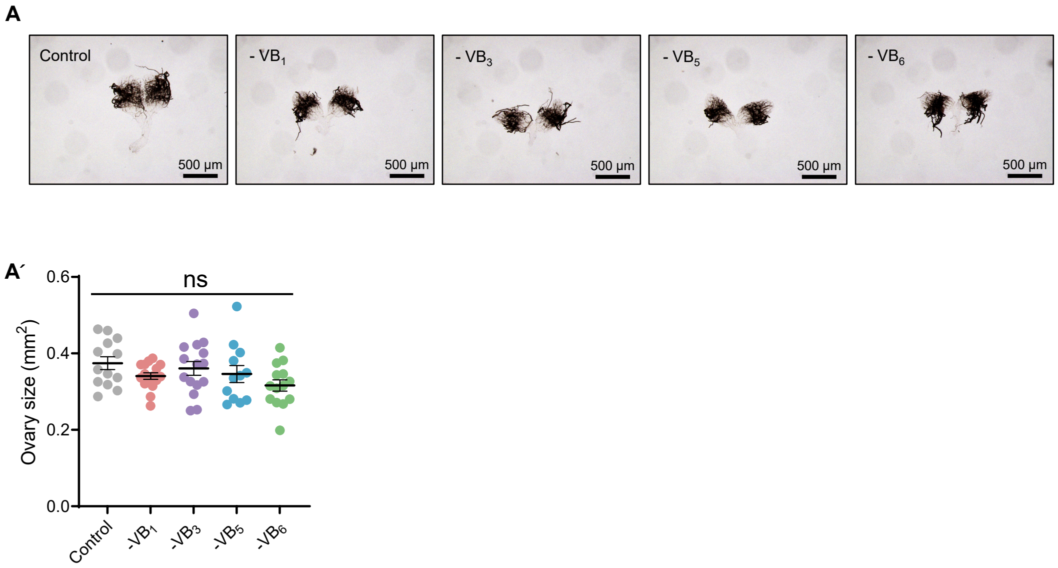

Supplementary Figure 2. The deprivation of vitamin B1, B3, B5, or B6 from the 100N50S FLYaa diet for a duration of 14 days had no significant effects on ovary morphology (A) and ovary size (A´) in *ovo*^{D1} infertile mutant females. n = 12 - 16 ovaries per treatment. Ovary size differences were assessed by one-way ANOVA followed by Tukey's multiple comparison.

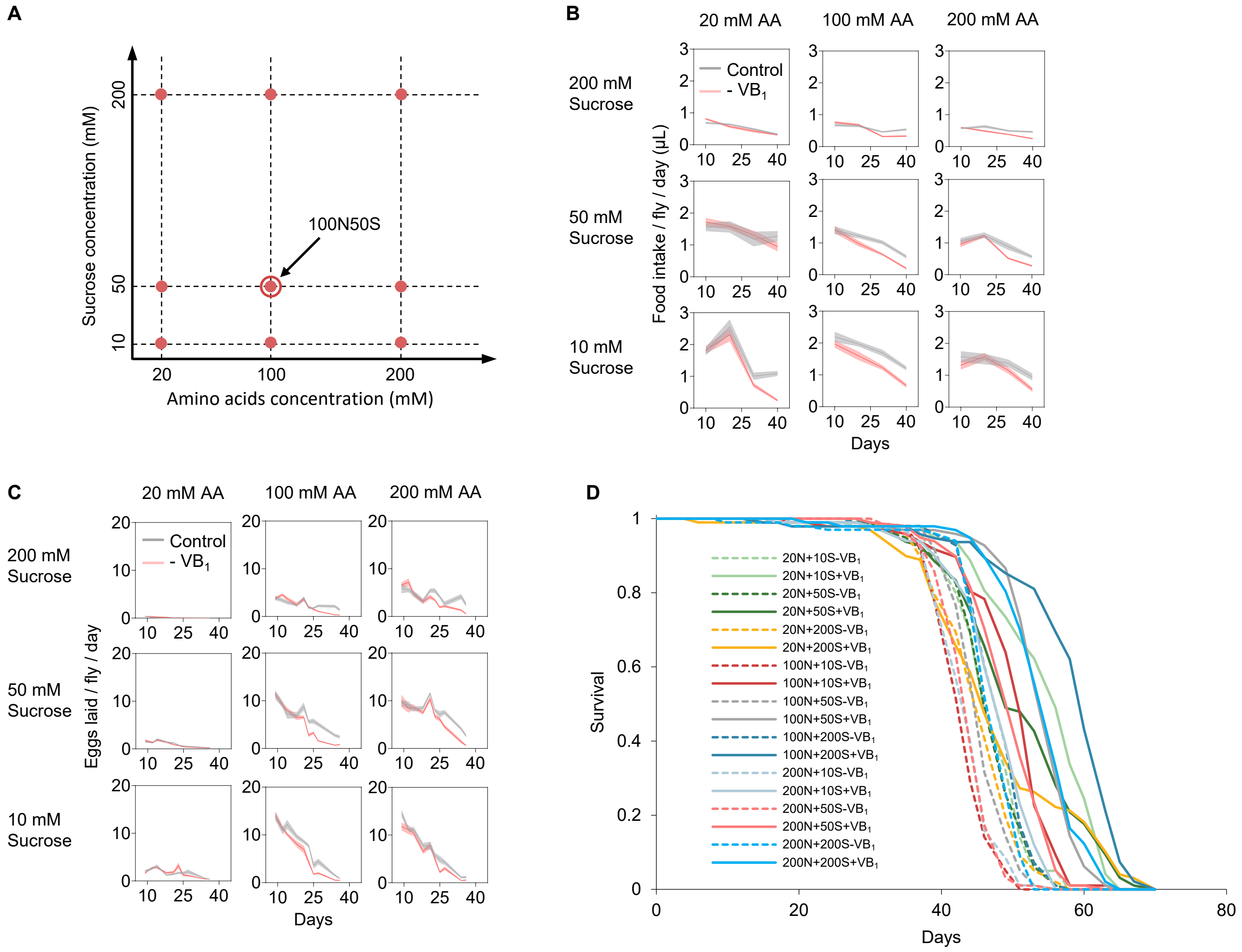

Supplementary Figure 3. The effect of VB_1 deprivation on food intake, fecundity and lifespan of wild-type *Dahomey* females in 9 diets with varying sucrose or amino acid concentrations. (A) Experimental diets used are indicated by red dots. 100N50S FLYaa medium was used as control diet. (B) Food intake from day 10 to day 40. Data are given as average food intake per female per day ± SEM. (C) eggs laid from day 9 to day 36. Data are given as mean number of eggs laid per female per day ± SEM. (D) Lifespan curves of wild-type *Dahomey* females in 9 diets with or without VB_1. (n = 100 flies per treatment for lifespans and n = 10 biological replicates for food intake and egg laying in all trials. See statistical analysis of lifespan data in Supplementary Table 4)

1.2 Supplementary Tables

Supplementary Table 1. Composition of experimental medium.

			FLYaa control diet (Amount per liter)	9 FLYaa diets with varying sucrose or amino acid contents (Amount per liter)								
	Ingredient	Stock	100N50S	20N10S	20N50S	20N200S	100N10S	100N50S	100N200S	200N10S	200N50S	200N200S
	Agar		7 g	7 g	7 g	7g	7 g	7 g	7g	7 g	7g	7 g
Sugar	Sucrose		17.12 g	3.424g	17.12g	68.48g	3.424g	17.12g	68.48g	3.424g	17.12g	68.48g
Amino acids (Low solubility)	l-isoleucine		0.56 g	0.112g	0.112g	0.112g	0.56g	0.56g	0.56g	1.12g	1.12g	1.12g
	l-leucine		1.02 g	0.204g	0.204g	0.204g	1.02g	1.02g	1.02g	2.04g	2.04g	2.04g
	l-tyrosine		0.46 g	0.092g	0.092g	0.092g	0.46g	0.46g	0.46g	0.92g	0.92g	0.92g
Add 670ml ddH$_2$O, bring to a boil and allow to cool to 60 °C before adding the following ingredients.												
Buffer		10×:<br>30 ml/l glacial acetic acid<br>30 g/l KH_2PO_4<br>10 g/l $NaHCO_3$	100 ml	100 ml	100 ml	100 ml	100 ml	100 ml	100 ml	100 ml	100 ml	100 ml
Metal ions	$CaCl_2$	1,000×: 250 g/l	1 ml	1 ml	1 ml	1 ml	1 ml	1 ml	1 ml	1 ml	1 ml	1 ml
	$MgSO_4$	1,000×: 250 g/l	1 ml	1 ml	1 ml	1 ml	1 ml	1 ml	1 ml	1 ml	1 ml	1 ml
	$CuSO_4$	1,000×: 2.5g/l	1 ml	1 ml	1 ml	1 ml	1 ml	1 ml	1 ml	1 ml	1 ml	1 ml
	$FeSO_4$	1,000×: 25 g/l	1 ml	1 ml	1 ml	1 ml	1 ml	1 ml	1 ml	1 ml	1 ml	1 ml
	$MnCl_2$	1,000×: 1 g/l	1 ml	1 ml	1 ml	1 ml	1 ml	1 ml	1 ml	1 ml	1 ml	1 ml
	$ZnSO_4$	1,000×: 25 g/l	1 ml	1 ml	1 ml	1 ml	1 ml	1 ml	1 ml	1 ml	1 ml	1 ml
Sterol	Cholesterol	20 mg/ml in EtOH	15 ml	15 ml	15 ml	15 ml	15 ml	15 ml	15 ml	15 ml	15 ml	15 ml
Amino acids	Essential amino acid stock solution (High solubility)	26.92 g/l l-arginine<br>10.79 g/l l-histidine<br>22.53 g/l l-lysine(HCl)<br>9.96 g/l l-methionine<br>16.65 g/l l-phenylalanine<br>18.27 g/l l-threonine<br>5.3 g/l l-tryptophan<br>19.81 g/l l-valine	30.26 ml	6.052ml	6.052ml	6.052ml	30.26ml	30.26ml	30.26ml	60.52ml	60.52ml	60.52ml
	Nonessential amino acid stock solution (High solubility)	18.18 g/l l-alanine<br>16.99 g/l l-asparagine<br>19.34 g/l l-aspartic acid<br>18.5 g/l l-glutamine	30.26 ml	6.052ml	6.052ml	6.052ml	30.26ml	30.26ml	30.26ml	60.52ml	60.52ml	60.52ml

		12.66 g/l glycine										
		16.14 g/l l-proline										
		22.74 g/l l-serine										
	Sodium glutamate stock solution	100 g/l sodium glutamate	7.59 ml	1.518ml	1.518ml	1.518ml	7.59ml	7.59ml	7.59ml	15.18ml	15.18ml	15.18ml
	l-cysteine HCl stock solution	50 g/l l-cysteine HCl	3.41 ml	0.682ml	0.682ml	0.682ml	3.41ml	3.41ml	3.41ml	6.82ml	6.82ml	6.82ml
Vitamins	thiamine	476×: 0.67 g/l	2.1ml	2.1ml	2.1ml	2.1ml	2.1ml	2.1ml	2.1ml	2.1ml	2.1ml	2.1ml
	riboflavin (Low solubility)	47.6×: 0.033 g/l	21ml	21ml	21ml	21ml	21ml	21ml	21ml	21ml	21ml	21ml
	nicotinic acid	476×: 3.99 g/l	2.1ml	2.1ml	2.1ml	2.1ml	2.1ml	2.1ml	2.1ml	2.1ml	2.1ml	2.1ml
	Ca pantothenate	476×: 5.16 g/l	2.1ml	2.1ml	2.1ml	2.1ml	2.1ml	2.1ml	2.1ml	2.1ml	2.1ml	2.1ml
	pyridoxine	476×: 0.83 g/l	2.1ml	2.1ml	2.1ml	2.1ml	2.1ml	2.1ml	2.1ml	2.1ml	2.1ml	2.1ml
	biotin	476×: 0.07 g/l	2.1ml	2.1ml	2.1ml	2.1ml	2.1ml	2.1ml	2.1ml	2.1ml	2.1ml	2.1ml
	folic acid	1,000×: 0.5 g/l	1 ml	1 ml	1 ml	1 ml	1 ml	1 ml	1 ml	1 ml	1 ml	1 ml
Other nutrients		125×:	8 ml	8 ml	8 ml	8 ml	8 ml	8 ml	8 ml	8 ml	8 ml	8 ml
		6.25 g/l choline chloride										
		0.63 g/l myo-inositol										
		8.13 g/l inosine										
		7.5 g/l uridine										
Preservatives	Propionic acid		6ml	6ml	6ml	6ml	6ml	6ml	6ml	6ml	6ml	6ml
	Nipagin	100 g/l methyl 4-hydroxybenzoate in 95% EtOH	15 ml	15 ml	15 ml	15 ml	15 ml	15 ml	15 ml	15 ml	15 ml	15 ml
Add ddH_2O to bring the volume to 1 liter												

Note: 20N, 100N and 200N represent amino acids concentration of 20 mM, 100 mM and 200 mM, respectively. 10S, 50S and 200S represent sucrose concentration of 10 mM, 50 mM and 200 mM, respectively.

Supplementary Table 2. Lifespan data for Figure 1B.

Dahomey females									
	Control	-All VBs	$-VB_1$	$-VB_2$	$-VB_3$	$-VB_5$	$-VB_6$	$-VB_7$	$-VB_9$
Number of death	198	197	197	197	198	198	195	197	203
median lifespan (d)	57	26.5	42.5	57	52	26.5	40	54.5	57
log rank test (vs control)		1.49E-86	6.16E-27	0.34374	0.00161	7.13E-91	2.41E-10	0.03319	0.76108
Dah,ovo^{D1} females									
	Control	-All VBs	$-VB_1$	$-VB_2$	$-VB_3$	$-VB_5$	$-VB_6$	$-VB_7$	$-VB_9$
Number of death	100	94	101	102	99	99	96	96	98
median lifespan (d)	66	42.5	61.5	64	64	57	54.5	64	58
log rank test (vs control)		1.16E-42	7.43E-05	0.26835	0.40766	1.06E-06	5.60E-10	0.43186	3.85E-08

Supplementary Table 3. Lifespan data for Supplementary Figure 1.

Dahomey females									
	Control	-All VBs	-VB_1	-VB_2	-VB_3	-VB_5	-VB_6	-VB_7	-VB_9
Number of death	100	101	99	96	100	98	99	102	96
median lifespan (d)	53.0	29.5	41.0	52.0	48.0	29.5	36.5	46.0	53.0
log rank test (vs control)		6.80E-45	4.51E-33	0.29129	6.28E-06	2.67E-41	1.14E-11	0.00041	0.77090
Dah,ovo^{D1} females									
	Control	-All VBs	-VB_1	-VB_2	-VB_3	-VB_5	-VB_6	-VB_7	-VB_9
Number of death	101	98	97	98	97	94	100	99	99
median lifespan (d)	62.0	43.5	57.5	62.0	64.5	55.0	50.5	64.5	57.5
log rank test (vs control)		3.26E-42	1.99E-11	0.05647	0.14986	4.75E-05	1.19E-09	0.68949	6.69E-08

Supplementary Table 4. Lifespan data for Supplementary Figure 3D.

		20N10S	20N10S	20N50S	20N50S	20N200S	20N200S	100N10S	100N10S	100N50S	100N50S	100N200S	100N200S	200N10S	200N10S	200N50S	200N50S	200N200S	200N200S
Number of death		100	98	98	96	98	99	99	97	98	97	98	95	95	100	98	98	100	97
median lifespan (d)		45.0	54.5	45.0	47.5	45.0	45.0	43.0	50.0	45.0	54.5	47.5	59.0	43.0	47.5	43.0	49	47.5	54.5
	20N10S-VB_1		6.38E-18	0.701593	2.51E-07	0.04403	0.034584	1.45E-10	1.94E-06	0.002576	1.64E-19	0.290922	4.92E-29	9.25E-08	0.208139	1.86E-08	0.000426	0.590375	2.57E-18
	20N10S+VB_1			4.48E-19	0.09847	2.38E-21	0.006273	1.30E-30	2.14E-09	3.81E-24	0.007039	6.09E-17	2.34E-05	7.47E-28	1.67E-16	2.92E-28	1.39E-11	1.63E-20	0.055946
	20N50S-VB_1				6.43E-08	0.119845	0.024001	2.83E-10	1.89E-07	0.004398	4.05E-21	0.137918	8.95E-30	2.52E-07	0.08165	8.01E-08	5.65E-05	0.6161	6.98E-20
	20N50S+VB_1					7.30E-10	0.198371	1.48E-17	0.013203	1.54E-11	0.78955	3.92E-06	8.30E-07	2.76E-15	5.38E-06	3.27E-15	0.00153	2.25E-08	0.530754
	20N200S-VB_1						0.001209	2.89E-05	2.91E-10	0.392337	4.69E-24	0.001883	3.86E-31	0.001668	0.0018	0.000922	2.45E-07	0.123408	1.11E-22
	20N200S+VB_1							7.31E-08	0.850206	0.000744	0.03734	0.193117	2.17E-07	1.81E-06	0.159509	1.93E-06	0.68087	0.054252	0.021064
	100N10S-VB_1								1.54E-23	2.05E-05	5.00E-38	2.54E-16	1.46E-36	0.243637	1.74E-14	0.206067	3.74E-20	3.65E-13	2.65E-36
	100N10S+VB_1									1.54E-14	7.53E-07	1.64E-05	7.24E-21	8.12E-20	0.000142	5.39E-21	0.206947	2.31E-10	7.13E-07
log rank test	100N50S-VB_1										3.99E-30	3.57E-06	4.65E-33	0.003957	4.19E-06	0.002318	5.95E-11	0.002921	4.08E-28
	100N50S+VB_1											8.14E-19	1.13E-11	2.26E-34	5.76E-17	9.26E-36	1.87E-09	2.74E-26	0.535155
	100N200S-VB_1												3.59E-28	4.07E-12	0.683875	1.88E-13	0.003589	0.023066	1.78E-17
	100N200S+VB_1													9.38E-35	1.29E-27	7.64E-35	5.06E-23	5.54E-31	1.68E-09
	200N10S-VB_1														4.13E-11	0.982829	1.99E-16	6.10E-09	1.06E-32
	200N10S+VB_1															5.53E-12	0.011654	0.008542	4.65E-16
	200N50S-VB_1																1.86E-17	3.81E-10	8.82E-34
	200N50S+VB_1																	9.26E-07	2.14E-09
	200N200S-VB_1																		5.97E-24
	200N200S+VB_1																		

Note: 20N, 100N and 200N represent amino acids concentration of 20 mM, 100 mM and 200 mM, respectively. 10S, 50S and 200S represent sucrose concentration of 10 mM, 50 mM and 200 mM, respectively.
